# Diseases Common to Man and Animals1Read before the Haverhill Medical Club, October, 1900.

**Published:** 1900-12

**Authors:** John M. Parker

**Affiliations:** Haverhill, Mass.


### DISEASES COMMON TO MAN AND ANIMALS.[1]

By John M. Parker, D.V.S.,
HAVERHILL, MASS.

The diseases common to man and animals, or, rather, to be more correct, to man and the lower animals, are numerous. The relationship between the various species of animals is a close one, more so, perhaps, than one might think from a mere casual glance at the subject. "There is but one animal kingdom," says Wesley Mills, "governed by the same natural laws, applicable alike to man and his fellow-creatures, lower in some respects in the scale,

[1] Read before the Haverhill Medical Club, October, 1900.

but sharing with him liability to disease and death." "There is only one science and art of medicine, and all the various bodies of workers in this vast field should form but different battalions of one great army, fighting for the prolongation of vigorous life and the mitigation of pain in every quarter to which the power of medicine can reach."

The diseases common to man and the lower animals may be roughly divided into contagious and infectious diseases, and parasitic diseases. Among the former, perhaps, the most important are anthrax, diphtheria, glanders, rabies, tuberculosis, and others; among the parasitic diseases may be mentioned trichina, measles, or cysticercus cellulosa, a larval form of the tænia solium of man, and others.

*Anthrax.* Anthrax in cattle is a disease that is practically unknown in New England, although not uncommon in New York State, Pennsylvania, Kansas, Minnesota, Nebraska, and many other States in the West and South. It appears in man usually from the handling of carcasses and hides of infected animals, and, except when carried from place to place by hides or hay and grasses, it is generally restricted to certain farms or localities.

Stagnant, undrained lands and certain seasons, when heavy rains occur, after hot, dry, warm weather, play an important part in its spread. Especially is this true if the carcass of an animal dying from anthrax is buried near the surface, where the conditions are favorable to spore-development. Then the bacilli, having rapidly increased in the body, or the formation of spores (which may remain capable of development for years) having occurred, the land is flooded with water, and the spores are carried to the surface, the grass becomes infected, and so in time the infected hay is carried away to spread infection to some other locality.

An animal affected with anthrax usually develops a high fever, sometimes as high as 106° or 107° F., and death follows in from twelve to twenty-four hours, or the disease may take a subacute form, and œdematous swellings may appear under the skin and in various parts of the body. Death usually supervenes in a few days' time.

On post-mortem examination the spleen is usually much enlarged and black in color. In the thoracic and abdominal cavities there is usually considerable serous exudations. The only positive method of diagnosis is the microscopical examination for anthrax bacilli. Treatment, as a rule, is of little avail. Preventive treatment consists in draining and turning up the land; in destroying the carcasses by burning, when possible, or destroying them by quicklime, or chloride of lime, and burying them deeply.

When anthrax appears in any locality preventive inoculation should be practised on the cattle on that farm and in the neighborhood. In France, where there is livestock insurance, preventive inoculation is required by the insurance companies.

*Diphtheria.* Diphtheria in man and the lower animals has not as yet been positively proven to be identical. Many cases of diphtheritic exudate are found in the domestic animals, but so far the presence of the Klebs-Löffler bacillus has not been positively demonstrated. It would seem, however, that to a large extent this may be due to the absence of observation and careful clinical, microscopical, and bacteriological examination rather than to the absence of the bacillus.

In this connection an interesting series of cases has been reported in a paper read by Dr. Winchester, of Lawrence, before the A. V. M. A. in 1896. He reports a series of cases that occurred in his practice. In all, six cows died from a disease of the larynx characterized by difficulty in swallowing, salivation, swellings, and discharge from the eyes and nose, hurried respiration, and great weakness. All showed similar symptoms.

Through the courtesy of Dr. Winchester it was my privilege to be present at two of the autopsies, and to make a microscopical examination of tissue from two of the cows. On the mucous membrane of the larynx there was a tough, adherent, yellowish-brown membrane. On microscopical examination sections showed a dense, fibrinous exudate, beneath which was a mass of small-celled proliferation. On staining with methylene-blue (Löffler's) solution, numerous short, thick, slightly-curved bacilli resembling the Klebs-Löffler bacillus were seen.

This was confirmed by Dr. H. C. Ernst, who writes as follows:

HARVARD MEDICAL SCHOOL, BACTERIOLOGICAL LABORATORY,
BOSTON, March 29, 1894.

DEAR DOCTOR: We have found in section of material from the trachea of the cow examined bacilli that, in size and general appearance, resemble very closely, if they are not identical with, the Klebs-Löffler bacillus.

Dr. Whitney wants to know if you are going to send the specimen for the museum? Very sincerely,

HAROLD C. ERNST.

This subject is one that needs thorough investigation, but in any event, whether the diseases are identical or not, whether the Klebs-Löffler bacillus is present or not, it should be remembered that diphtheria in some form is not uncommon in the lower animals, and that there is some danger of infection being carried from one person to another, especially by the smaller domestic animals like the cat and the dog.

*Glanders* is an insidious disease, most commonly found in the horse, and through the use of mallein (like tuberculosis) it will be found to be far more common in a latent form than has been generally supposed.

Glanders seems to be seriously increasing in this State. Since 1879, when the matter was first taken up by the State, and when there were forty-three glandered horses killed, the number has tremendously increased. There were 18 horses killed by the State for glanders in 1885; 75 were killed in 1886; there was an outbreak in the stables of the Boston Street Railway Company in 1887, when a number were killed, and a still larger number were tied up in quarantine. Each year the number has kept increasing. In 1894 there were 160, then 206, 341, 402, and 387, until in 1899 there were 543 cases reported. This is a serious state of affairs, although the increase is partly explained by the fact that greater care is taken in looking up cases and reporting them to the State officials.

Glanders is the most dangerous disease affecting the horse. Bovines are not generally susceptible. Probably, in the majority of cases, the disease is contracted through the respiratory organs, although infection through the digestive tract is not uncommon.

Some few years ago a series of cases came under my notice that were interesting and are worth recording. In a stable in the central part of the State fourteen horses were accommodated, seven in one end of the barn and seven in the other. The centre of the barn was open to the front, and the wagons were backed into this part. The doors by which the horses entered the stable opened off to the right and left from this shed. The wagon-pole and neck-yoke used by the horse originally infected were left standing beside the door by which the barn was entered, and, seemingly attracted by the discharge on the neck-yoke, all the horses at this end of the stable on their way in and out used to stop and lap the neck-yoke hanging on the pole beside the stable-door. Unfortunately, before any suspicion was aroused every horse at this end of the barn, with one single exception, had contracted the disease and were finally destroyed.

Inoculation through the digestive tract has also taken place in a series of experiments that will be referred to later. In the meantime it is enough to state that Professor Nocard, of Alfort, caused twelve horses to drink water in which a measured quantity of glanders culture had been placed, and all developed the disease.

The typical symptoms of glanders are a viscid, yellowish-gray, gluey discharge of unhealthy character, sometimes mixed with blood due to ulcerative destruction of small vessels. On the lower part of the nasal septum chancres with dentated, thickened edges and grayish bottom usually ultimately form. The submaxillary glands enlarge, and generally have a hard, circumscribed feel to the touch. As the disease develops the general nutrition appears to suffer. Unfortunately, however, typical symptoms are not always present. Many cases are obscure, and it is only on the most careful examination that evidence of glanders can be detected. These are by far the most dangerous, because they spread the disease while they themselves are undetected.

Farcy is simply a superficial manifestation of the same disease in which the lymphatics are involved. Tubercles are usually found on the lungs, but the lesions on post-mortem examination may be found in any of the organs of the body. Cases have frequently occurred in man, usually from direct contact with a glandered horse. About two years ago a case came under my observation where a man having charge of a stable where glandered horses were found developed farcy sores on his legs. He was under treatment for some time, and finally recovered. Recently a friend and his son both died from glanders contracted while caring for a horse under the impression that it was suffering from simple catarrh.

Diagnosis in suspicious cases can usually be confirmed by the mallein-test, which is similar to the tuberculin-test for tuberculosis. Guinea-pig inoculation is, as a rule, also satisfactory, the swelling of the testicles being characteristic.

Treatment of glanders in horses is forbidden in this State, the law providing that all horses suffering from glanders must be destroyed. Some interesting experiments, however, were carried on in the fall of 1896 by Nocard in conjunction with the War Department in France. Twelve horses were given water containing a measured quantity of the culture. From four to eight days afterward they were feverish, and about two weeks afterward all had contracted the disease, and all reacted to the mallein-test by both thermic and local reaction. A month later another injection of mallein was followed by a similar reaction, only less severe. On the third month five horses showed such marked clinical symptoms of glanders that they were destroyed, and in another month a sixth died.

The injections were kept up every month, and at the sixth month none of the remaining six animals were giving any reaction to the mallein-test. Eight months afterward four of the six were destroyed, and in all miliary tubercles were found in the various forms characteristic of glanders, but sterile. *The animals had recovered.* The remaining two horses were returned to their work in the army, where they will be carefully watched for future developments.

The experiments have since been repeated by Nocard with similar results, until he has reached the conclusion that complete absence of thermal reaction after its full development or after a gradual diminution *means death to the bacillus, means recovery.*

An interesting case in this same line is reported in the *Veterinary Review* for December, 1895, in a translation from the German. "The author has had occasion to treat with mallein a case of human glanders. This observation, unique at this day, brings him to the following conclusions :

"1. Mallein obtained by cultures gives the man affected with chronic glanders a reaction far less violent than in the horse. Two or three drops are sufficient, instead of 1 c.c., as required by the horse. The intensity of the reaction is proportionate to the dose, but goes on decreasing as the injections are repeated.

"2. Each injection gives rise to an œdematous and painful swelling, which rapidly disappears.

"3. During the days following the injection the temperature often drops to a very low degree, and stays there for twenty-four to forty-eight hours, without any objective or subjective symptoms.

"4. The reaction is accompanied with very abundant polyuria, even with profuse perspiration. The urine is almost always thoroughly alkaline.

"5. The action of the injections of mallein upon the local manifestation of glanders has proved very efficacious.

"The author has also attempted to establish the curative value of mallein on guinea-pigs, dogs, and horses.

"In the first it is mallein obtained with the aid of the serum of bovines which seemed to have the greatest efficacy. A recovery has been obtained in a horse which so far has lasted a year."

These experiments have been so successful that further trial and investigation would seem to be called for, and it seems to me that some work in this direction should be done by our State authorities.

(To be continued.)

